# Efficacy and safety of finerenone in chronic kidney disease and type 2 diabetes patients: a systematic review and meta-analysis

**DOI:** 10.1097/MS9.0000000000001180

**Published:** 2023-08-16

**Authors:** Farah Yasmin, Muhammad Aamir, Hala Najeeb, Abdul Raafe Atif, Abdul Hannan Siddiqui, Muhammad Nadeem Ahsan, Abdul Moeed, Syed Hasan Ali, Haya Muhammad Tahir, Muhammad Sohaib Asghar

**Affiliations:** a Department of Internal Medicine, Dow Medical College, Dow University of Health Sciences; b Department of Internal Medicine, Jinnah Sindh Medical University; c Department of Nephrology and Dialysis Unit, Dow University of Health Sciences-Ojha Campus, Karachi, Pakistan; d Department of Cardiovascular Medicine, Lehigh Valley Health Network, Allentown, Pennsylvania; e Division of Nephrology and Hypertension, Mayo Clinic - Rochester, Minnesota, USA

**Keywords:** chronic kidney disease (CKD), finerenone, mineralocorticoid receptor antagnosits (MRA), type 2 diabetes, meta-analysis

## Abstract

**Background and objectives:**

The incidence of morbidity and mortality in patients with type 2 diabetes mellitus is substantially correlated with cardiovascular disease and chronic kidney disease. The current guidelines recommend the use of renin-angiotensin system blockers, but recent studies probed into the effects of finerenone to mitigate the risk of cardiorenal events. This meta-analysis was performed to demonstrate the effects of finerenone on cardiorenal events, comprising cardiovascular mortality, heart failure, change in estimated glomerular filtration rate, and serum potassium levels.

**Methods:**

After screening with our eligibility criteria, 350 articles were identified with an initial literature search on multiple databases, including PubMed, Science Direct, and Cochrane Central. Seven randomized controlled trials with a total of 15 462 patients (*n*=8487 in the finerenone group; *n*=6975 in the control group) were included.

**Results:**

Patients receiving finerenone were at a reduced risk for cardiovascular mortality [HR: 0.84 (0.74, 0.95)], heart failure [OR: 0.79 (0.68, 0.92)], decrease in estimated glomerular filtration rate by 40% [OR: 0.82 (0.74, 0.91)] and by 57% [OR: 0.70 (0.59, 0.82)]; and a higher incidence of moderate hyperkalemia [OR: 2.25 (1.78, 2.84)].

**Conclusion:**

Finerenone, owing to its better mineralocorticoid affinity, and a much lower risk of adverse effects, promises to be a much better alternative than other renin-angiotensin system blockers available for the treatment of chronic kidney disease patients with type 2 diabetes. Further trials should be conducted to provide more definitive evidence to assess the safety and efficacy of finerenone compared to spironolactone and eplerenone.

## Introduction

Highlights

- This meta-analysis was performed to see the effects of finerenone on cardiorenal events, including cardiovascular mortality.
- Further trials should be conducted to provide more definitive evidence to assess the safety and efficacy of finerenone compared to spironolactone and eplerenone.

The incidence of morbidity and mortality in patients with type 2 diabetes (T2D) is substantially correlated with cardiovascular disease (CVD), escalating in conjunction with chronic kidney disease (CKD)^1^. With T2D being the primary cause of CKD^2^, current guidelines recommend the use of renin-angiotensin system blocker^3,4^ to limit hypertension for the treatment of CKD in patients with T2D. Based on this information, recent studies^5,6^ probed into the effects of finerenone to mitigate the risk of cardiorenal events. In preclinical studies, finerenone has been established as a highly selective third-generation nonsteroidal mineralocorticoid receptor antagonist (MRA) instead of spironolactone and eplerenone, with more pronounced anti-inflammatory and antifibrotic effects^7–9^.

Finerenone has been documented to decrease urinary albumin-to-creatinine ratio, with significantly lower potassium levels observed compared to spironolactone^10^. Furthermore, finerenone was shown to be well-tolerated in CKD patients with T2D^10^. In a recent meta-analysis^11^, finerenone significantly reduced cardiovascular events whereas, no reduction in estimated glomerular filtration rate (eGFR) was seen. However, notable recent trials FIDELIO-CKD and FIGARO-CKD showed a significant reduction in eGFR decline and cardiovascular events. We conducted this meta-analysis to help reconcile the variable results and form a conclusive, well-powered assessment of the effect of finerenone in the management of CKD in T2D patients with or without baseline CVD.

## Methods

This meta-analysis was performed per the Preferred Reporting Items for Systematic Review and Meta-Analyses (PRISMA) guidelines^12^ (Supplemental Digital Content 1, http://links.lww.com/MS9/A211) and follows the framework laid out by the Cochrane Collaboration (Supplementary Figure 1, Supplemental Digital Content 2, http://links.lww.com/MS9/A212). The work has been reported in line with AMSTAR (Assessing the methodological quality of systematic reviews) Guidelines^13^ (Supplemental Digital Content 3, http://links.lww.com/MS9/A213).

### Data sources and search strategy

We systematically searched PubMed (Medline), Cochrane Central, and Science Direct from inception to November 2021 with no filters or time and language restriction. A detailed search string was created using Medical Subject Headings (MeSH terms) and keywords including [‘finerenone’] AND [‘chronic kidney disease’ OR ‘diabetic kidney disease’]. The detailed search strategy used in each database is presented in Supplementary Table 1 (Supplemental Digital Content 2, http://links.lww.com/MS9/A212).

### Study selection

Articles retrieved from the literature search were exported to Endnote Reference Library (Version X7.5; Clarivate Analytics) software, where the duplicates were identified and removed. The remaining articles were then thoroughly reviewed by independent reviewers (A.H.S. and H.N.), ensuring that the selected articles met the predefined eligibility criteria. The original articles were screened and extracted for further review. Studies were selected based on the following eligibility criteria: randomized controlled trials; adult patients over 18 years of age with T2D and CKD; with or without CVD at baseline; CKD defined by all three definitions (albuminuria, eGFR, and creatinine clearance). Data of study and patient characteristics and cardiorenal events, namely cardiovascular mortality, HF, change in eGFR, and serum potassium were extracted. Any observational study, case report, or reviews were excluded at the time of screening.

### Data extraction

Data was extracted by two researchers (A.H.S. and H.N.) according to the following fields of interest: author, year of publication, study design, phase of trial, clinical trial identification code, sample size, age of participants, intervention and control therapy, percent of males in the study, proportion of participants with CKD or CVD, and follow-up time. Table 1 shows the baseline and study characteristics of all included trials. Additionally, effect sizes and ratios (OR, HR, and RR) were extracted for patient related outcomes of all-cause mortality, cardiovascular mortality, heart failure, decrease in eGFR by 40% from baseline, decrease in eGFR by 57% from baseline, any change in eGFR from baseline, moderate hyperkalemia, or mild hyperkalemia. Mild hyperkalemia was defined as a serum potassium greater than 5.5 mmol and moderate hyperkalemia was serum potassium greater than 6 mmol.

**Table 1:** Baseline and study characteristics

Study	Study name	Design	Drug	Control	Sample size	Follow-up (years)	Mean age (years)	Males (%)	Outcomes
Rossing ^14^	FIDELIO-DKD	Secondary analysis	Finerenone	Placebo	5674	2.6	65.6	3983 (70.2)	Cardiovascular mortality, heart failure, decrease in eGFR, hyperkalemia.
Rossing ^15^	FIDELIO-DKD	Subgroup analysis	Finerenone	Placebo	5674	2.6	65.6	3983 (70.2)	
Filippatos ^16^	FIDELIO-DKD	Secondary analysis	Finerenone	Placebo	5674	2.6	65.6	3983 (70.2)	
Filippatos ^17^	FIDELIO-DKD	Secondary analysis	Finerenone	Placebo	5674	2.6	65.6	3983 (70.2)	
Agarwal ^18^	FIDELIO-DKD	Secondary analysis	Finerenone	Placebo	5674	2.6	65.6	3983 (70.2)	
Bakris ^5^	FIDELIO-DKD	RCT	Finerenone	Placebo	5674	2.6	65.6	3983 (70.2)	
Pitt ^6^	FIGARO-DKD	RCT	Finerenone	Placebo	7352	3.4	64.1	5105 (69.4)	Cardiovascular mortality, heart failure, decrease in eGFR, hyperkalemia.
Katayama ^19^	ARTS-DN Japan	RCT	Finerenone	Placebo	96	N/R	62.9	77 (80.2)	Change in eGFR.
Filippatos ^20^	ARTS-HF	RCT	Finerenone	Eplerenone	1055	N/R	71.2	816 (77.3)	Cardiovascular mortality, heart failure, decrease in eGFR, hyperkalemia.
Sato ^21^	ARTS-HF Japan	RCT	Finerenone	Eplerenone	72	0.33	73.1	53 (73.6)	Heart failure, decrease in eGFR, hyperkalemia.
Bakris ^22^	ARTS-DN	RCT	Finerenone	Placebo	821	N/R	64.2	639 (78)	Change in eGFR, hyperkalemia.
Pitt ^10^	ARTS	RCT	Finerenone	Placebo and Spironolactone	392	0.13	72.1	312 (79.6)	Heart failure, change in eGFR, hyperkalemia.

### Study quality assessment

The risk of bias was assessed using the Revised Cochrane Risk of Bias tool (RoB-2) for randomized controlled trials (RCTs)^23^ by two independent investigators (A.H.S. and H.N.). Studies were evaluated for the robustness of their protocol, methods, and outcomes. The updated version of the tool has five domains to check the biasness: (D1) randomization process; (D2) deviations from intended interventions; (D3) missing outcome data; (D4) measurement of the outcome; and (D5) selection of the reported results. To check for bias, assessors answered various questions regarding each domain. Any trial with a domain at a ʻhigh riskʼ of bias, was to be judged as to have a high risk of bias overall. Similarly, if a trial was going to have ʻsome concernsʼ in one or more domains, it was going to be judged to have some concerns overall.

### Statistical analysis

Statistical analysis was performed using Review Manager (version 5.3). Using a random-effects model, categorical variables were pooled using odds ratios (ORs) and corresponding 95% CIs, whereas continuous outcomes were pooled to estimate a weighted mean difference with a 95% CI. This meta-analysis reports a pooled effect of ORs and weighted mean differences using the generic-inverse variance and continuous outcome functions with a random-effects model. Each effect size was reported on a log scale, and the 95% CI was converted to standard error to normalize the data distribution. At every instance, a *P*-value of <0.05 was considered significant.

Heterogeneity was assessed using the Higgins *I* ^2^ test^19^, with *I* ^2^ greater than 75% being considered significant. A value of 25% was considered low heterogeneity while 25–75% was moderate heterogeneity. To explore the effect of each study on the pooled estimate, a sensitivity analysis was performed with studies that had outcomes with a high percentage of heterogeneity.

## Results

### Study selection and characteristics

The initial search revealed 402 articles, of which 273 records were screened for a detailed evaluation, as shown in Supplementary Figure S1 (Supplemental Digital Content 2, http://links.lww.com/MS9/A212). Following the exclusion criteria, articles were rejected due to incorrect intervention, patient population, and irrelevant outcomes. Seven RCTs with a total of 15 462 patients (finerenone *n*=8487; control *n*=6975) were included in the meta-analysis^5,6,10,14,20–22^. Five studies which provided additional information to the RCTs, such as secondary or subgroup analysis, were included in the quantitative analysis^14–18^.

Baseline study and patient characteristics of all included studies are presented in Table 1. The overall mean follow-up time was 1.6 years, with the maximum mean follow-up time being 3.4 years for the FIGARO-DKD trial. The mean age seen overall was 67.6 years. Four out of seven RCTs compared the effect of finerenone with a placebo group^5,6,14,21^, two studies compared the effect of finerenone with eplerenone^20,22^, and one study had patients taking either a placebo or spironolactone in the comparator group^10^.

#### CV mortality and heart failure

**Figure 1:**
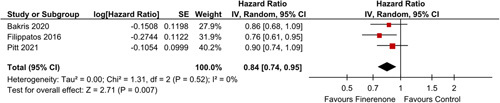
CV Mortality in patients treated with finerenone or control. Red squares and their corresponding lines are the point estimates and 95% CI per study. Black diamonds represent the pooled effect estimate.

**Figure 2:**
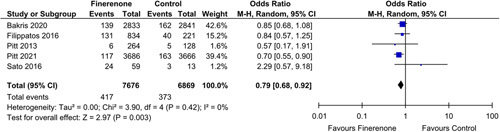
Heart Failure in patients treated with finerenone or control. Blue squares and their corresponding lines are the point estimates and 95% CI per study. Black diamonds represent the pooled effect estimate.

Three out of twelve studies gave cardiovascular mortality as an outcome, while five out of twelve gave heart failure as an outcome. Analysis of patients receiving finerenone showed a reduced risk for cardiovascular mortality [HR: 0.84 (0.74, 0.95); *P*: 0.007; *I*²=0%] (Fig. 1), along with a reduced incidence of heart failure [OR: 0.79 (0.68, 0.92); *P*: 0.003; *I*²=0%] (Fig. 2), when compared to the control group.

#### eGFR

**Figure 3:**
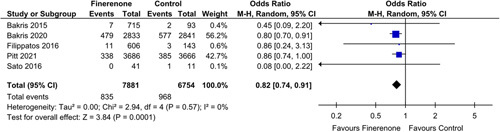
Decrease in eGFR by 40% in patients treated with finerenone or control. Blue squares and their corresponding lines are the point estimates and 95% CI per study. Black diamonds represent the pooled effect estimate.

**Figure 4:**
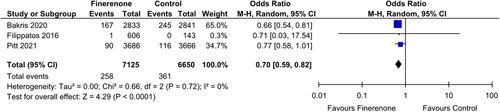
Decrease in eGFR by 57% in patients treated with finerenone or control. Blue squares and their corresponding lines are the point estimates and 95% CI per study. Black diamonds represent the pooled effect estimate.

A significant decrease of risk in eGFR decline by 40% from baseline was shown by five studies [OR: 0.82 (0.74, 0.91); *P*: 0.0001; *I*²=0%] (Fig. 3). Additionally, patients prescribed to finerenone experienced a decrease risk in eGFR decline from baseline by 57% [OR: 0.70 (0.59, 0.82); *P*<0.0001 ; *I*²=0%] (Fig. 4), as was seen in three out of twelve studies.

### Subgroup analysis

**Figure 5:**
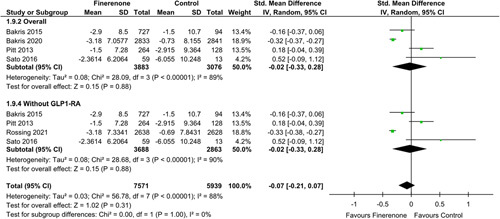
Change in eGFR (ml/min/1.73m2) in patients treated with finerenone or control. Green squares and their corresponding lines are the point estimates and 95% CI per study. Black diamonds represent the pooled effect estimate.

A subgroup analysis was conducted in which RCTs were sub-grouped, assessing their effectiveness individually. A subgroup analysis performed for overall change in eGFR from the baseline category having patients who did not use GLP1-RA at baseline revealed no significant association with finerenone; SMD: [−0.02 (−0.33, 0.28); *P*: 0.88; *I*²=90%]^4–7^ (Fig. 5).

#### Hyperkalemia

**Figure 6:**
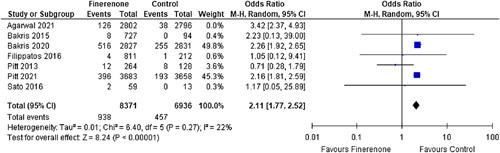
Moderate Hyperkalemia in patients treated with finerenone or control. Blue squares and their corresponding lines are the point estimates and 95% CI per study. Black diamonds represent the pooled effect estimate.

Seven out of twelve articles had moderate hyperkalemia as an outcome of interest. Finerenone was associated with a higher incidence of moderate hyperkalemia [OR: 2.25 (1.78, 2.84); *P*<0.00001; *I*²=49%] (Fig. 6) when compared to the control group.

**Figure 7:**
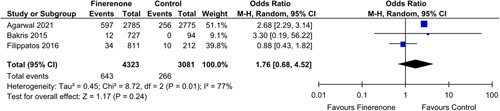
Mild Hyperkalemia in patients treated with finerenone or control. Blue squares and their corresponding lines are the point estimates and 95% CI per study. Black diamonds represent the pooled effect estimate.

Mild hyperkalemia was recorded by only three out of twelve studies; however, due to the very high heterogeneity seen in the data, it could not be accredited to the intervention drug [OR: 1.76 (0.68, 4.52); *P*: 0.24; *I*²=77%] (Fig. 7).

### Sensitivity analysis

A sensitivity analysis was performed after identifying and excluding RCTs with low-quality assessment scores or studies with different demographics and the number of patients in both, the intervention and control groups. For the moderate hyperkalemia outcome, excluding two studies (Agarwal 2021, Pitt 2013) which led to a decline in hetreogenity, from *I*²=49% to *I*²=0%; however, with no change in the outcome [OR: 2.21 (1.96, 2.49); *P*<0.00001) (Supplementary Figure S2, Supplemental Digital Content 2, http://links.lww.com/MS9/A212).

### Quality assessment and publication bias

**Figure 8:**
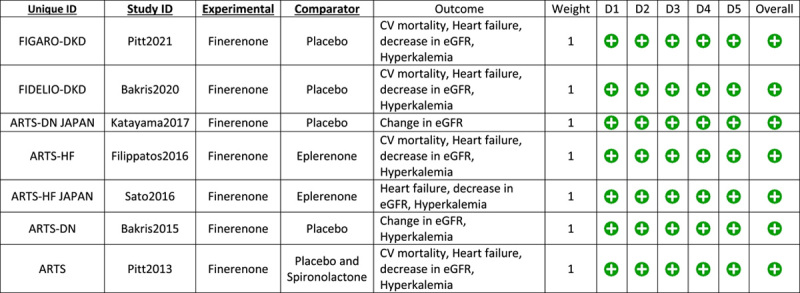
Risk of Bias Assessment.

A quality assessment was performed for the seven original RCT’s. An RCT was considered at an overall low risk of bias when it had a complete methodology, following all rules and regulations of good ethical practices. The analysis was done on an intention-to-treat model. All seven RCT’s reported a low risk of bias overall and in all individual components as well (Fig. 8). A detailed assessment is included in the Supplementary Material (Supplemental Digital Content 2, http://links.lww.com/MS9/A212).

## Discussion

The results of our meta-analysis suggest that finerenone use is associated with a lower incidence of heart failure events and cardiovascular mortality; a lower risk of a sustained decrease in eGFR, and an increased risk of moderate hyperkalemia in CKD patients with T2D when compared to patients in the control group.

Although MRA are effective in the treatment of CKD^24,25^, they are rarely used in these patients^26^. One of the possible explanations is that the use of the steroidal MRAs is associated with progesterone and androgen-dependent adverse effects^27^. Finerenone (BAY 94-8862), a novel, selective nonsteroidal MRA has better selectivity than spironolactone and a better affinity to mineralocorticoid receptors (MR) than eplerenone. Furthermore, finerenone has a significantly higher selectivity for MR than androgen receptors, progesterone receptors, and glucocorticoid receptors^28^.

Previous meta-analyses on the efficacy and safety of finerenone on the CKD patients with T2D have revealed contrasting findings regarding the change in eGFR in these patients. A meta-analysis by Fu *et al*.^11^ revealed that there was no significant difference in the change of eGFR of patients with CKD between the finerenone group and the placebo group. However, meta-analyses by Zhang *et al*.^29^ and Zheng *et al*.^30^ both found that patients with greater than or less than 40% reduction in eGFR from baseline were significantly lower in the finerenone group when compared to the placebo group. The FIGARO-DKD trial, a recent, double blinded, randomized, multicentre phase III study, compared the cardiovascular and kidney outcomes between the finerenone and the placebo group in patients with CKD and T2D. The inclusion of the FIGARO-DKD trial in the meta-analyses by Zhang *et al*. and Zheng *et al*. may explain this discrepancy in the eGFR results.

Although previous meta-analyses have been conducted on the efficacy and safety of finerenone on patients with T2D and CKD, they only included RCTs^11,29,30^. Our analysis also included five subgroup/secondary analyses of the RCTs in addition to the RCTs. A secondary analysis by Rossing *et al*.^16^ investigated the effect of Glucagon-like-peptide-1 receptor agonists (GLP-1RA) on the treatment effect of finerenone. Since GLP-1RA use is associated with better renal outcomes in patients with CKD and T2DM^31–33^, the study by Rossing *et al*. was included to perform a subgroup analysis of patients not using GLP-1RA to reduce any confounding bias.

The FIGARO-DKD trial was the first trial to demonstrate that an MRA can decrease or even prevent the development of heart failure in patients with CKD and T2D^34^. Another trial, FIDELIO-DKD, found that finerenone was associated with a lower risk of CKD progression and cardiovascular events in CKD patients with T2D^5^. These results are consistent with the results of our analysis. Furthermore, Filippatos *et al*., in a secondary analysis of the FIDELIO-DKD trial found that in patients with CKD and T2D, finerenone use reduced the risk of new onset atrial fibrillation or flutter.

In patients with CKD and T2D, the overall change in eGFR was similar in both the finerenone and the placebo group when eGFR was assessed as a continuous outcome. However, finerenone use was significantly associated with a lower risk of eGFR reduction by 40% and a lower risk of eGFR reduction by 57% when analyzed as a categorical variable. The discrepancy in the eGFR results when assessed as a categorical or continuous could be potentially explained by the large FIGARO-DKD trial not reporting eGFR as a continuous variable and therefore not included in the analysis of this outcome. These results suggest that finerenone use may delay CKD progression in T2D patients, which could be attributed to the direct effect of finerenone on the heart and vasculature due to mineralocorticoid receptor activation^8,35^. Finerenone use was shown to have a significantly lower decrease in eGFR when compared to spironolactone^10^. Since Glucagon-like-peptide-1 receptor agonists (GLP-1RA) use is independently associated with a change in eGFR^31,36^, a subgroup analysis excluding patients using GLP-1Ras was performed for the overall change in eGFR. The change in eGFR; however, remained nonsignificant with finerenone use.

A major adverse event of finerenone use, highlighted by our analysis, is hyperkalemia. This is due to the potassium-sparing effect of MRAs, which increases serum potassium concentration^37^. The increased risk of hyperkalemia associated with finerenone use when compared to placebo in CKD and T2D patients has been supported by previous meta-analyses^11,29,30^ and a safety post-hoc analysis of the FIDELIO-DKD trial by Agarwal *et al*.^38^. Although finerenone use was associated with a higher risk of hyperkalemia, discontinuation of the trial regimen due to hyperkalemia was still rare^5^. However, the mean increase in potassium concentration was found to be significantly lower in the finerenone group than in the spironolactone or eplerenone group^10,39,40^. Routine potassium monitoring and hyperkalemia management strategies are considered appropriate to manage the risk of hyperkalemia in CKD patients with T2D^38^.

Certain limitations must be kept in mind while interpreting the results of this study. First, there is a lack of uniformity among the control groups of the various studies included. While most trials compared the effect of finerenone to a placebo group, a few trials used eplerenone in the control group. Another limitation of the study is that the FIGARO-DKD trial and the FIDELIO-DKD trial provided almost 85% of the study cohort analyzed in this meta-analysis. The other trials were underpowered and did not add much to the statistical power of the two larger trials combined. In some results, the heterogeneity is very high. It seems not relevant to consider those results unless more studies are done to update the current meta-analysis in the future. Discrepancies exist in the end-point definitions, study designs, patient characteristics, and follow-up durations of the patients among the included studies, which can lead to possible clinical heterogeneity. Owing to a lack of studies, a visual inspection of the funnel plot could not be obtained to assess the possible publication bias. Therefore, well-powered RCTs are required to assess the existing clinical relevance and evidence of the efficacy of finerenone in CKD and T2D patients. Finally, while protocol registration is highly recommended before conducting a meta-analysis^41^, this update to the previous meta-analysis was not preregistered.

## Conclusion

Data from our analysis suggests that finerenone reduces the risk of heart failure and cardiovascular mortality in patients with T2D and CKD while also delaying the progression of CKD in these patients. Although a higher risk of hyperkalemia was observed with finerenone use compared to placebo, it was rarely severe enough to merit discontinuation of the trial regimen. Further trials should be conducted to provide more definitive evidence to assess the safety and efficacy of finerenone compared to spironolactone and eplerenone.

## Ethical approval

Not applicable.

## Consent

Not applicable.

## Sources of funding

This work is not supported by any sponsors. No funding required in this study.

## Author contribution

List of all Authors: F.Y.; M.A.; H.N.; A.R.A.; A.H.S.; M.N.A.; A.M.; S.H.A.; I.U.; M.S.A.

Corresponding Author: M.S.A.

This statement is to certify that all authors have seen and approved the manuscript being submitted, have contributed significantly to the work, attest to the validity and legitimacy of the data and its interpretation, and agree to its submission to the Annals of Medicine and Surgery.

We attest that the article is the Authors' original work, has not received prior publication and is not under consideration for publication elsewhere. We adhere to the statement of ethical publishing as appears in the journal statement on ethical standards in publishing scientific articles in the IJS

publishing group.

On behalf of all Co-Authors, the corresponding Author shall bear full responsibility for the submission.

Any changes to the list of authors, including changes in order, additions or removals will require the submission of a new author agreement form approved and signed by all the original and added submitting authors.

## Conflicts of interest disclosure

I undersign, certificate that I do not have any financial or personal relationships that might bias the content of this work.

## Research registration unique identifying number (UIN)

Registered with Research registry (UIN: reviewregistry1540).

## Guarantor

Muhammad Sohaib Asghar.

## Data availability statement

No new datasets generated.

## Provenance and Peer review:

Externally peer reviewed, not commissioned.

## Supplementary Material

SUPPLEMENTARY MATERIAL
