# Supplementary material for: Efficacy and safety of finerenone in chronic kidney disease and type 2 diabetes patients: a systematic review and meta-analysis
Source: Ann Med Surg (Lond). 2023 Aug 16;85(10):4973–80. doi: 10.1097/MS9.0000000000001180 (PMC10553111; doi:10.1097/MS9.0000000000001180)

**Online Supplementary**

**Table 1. Detailed search strategy in each database.**

| PubMed | (BAY 94–8862 OR finerenone) AND (CKD OR chronic kidney disease OR DKD OR type 2 diabetes OR T2D OR diabetic kidney disease OR albuminuria) |
| --- | --- |
| Cochrane Central | (BAY 94–8862 OR finerenone) AND (CKD OR chronic kidney disease OR DKD OR type 2 diabetes OR T2D OR diabetic kidney disease OR albuminuria) |

**Supplementary Figure 1. Prisma Flow Chart.**

**Supplementary Figure 2: Sensitivity analysis of Moderate hyperkalemia after excluding Agarwal 2015 and Pitt 2013.**
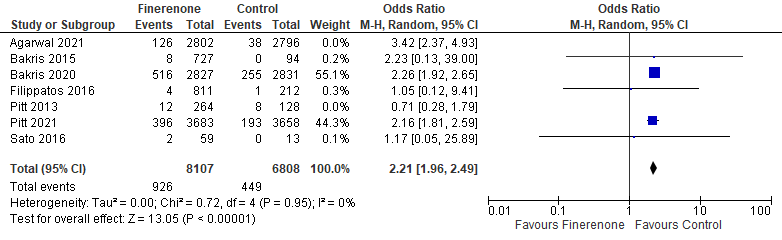

Supplement: SUPPLEMENTARY MATERIAL [file ms9-85-4973-s002.docx]
